# Spatiotemporal Dynamics of Avian Influenza: Understanding Avian Influenza Transmission via Mallard Migration Data

**DOI:** 10.1155/ipid/5555858

**Published:** 2025-12-11

**Authors:** Sena Mursel, Brian D. Davison, Thomas McAndrew, Bilal Khan, Paolo Bocchini

**Affiliations:** ^1^ Department of Civil and Environmental Engineering, Lehigh University, Bethlehem, Pennsylvania, USA, lehigh.edu; ^2^ Center for Catastrophe Modeling and Resilience, Lehigh University, Bethlehem, Pennsylvania, USA, lehigh.edu; ^3^ Department of Computer Science and Engineering, Lehigh University, Bethlehem, Pennsylvania, USA, lehigh.edu; ^4^ Department of Biostatistics and Health Data Science, Lehigh University, Bethlehem, Pennsylvania, USA, lehigh.edu

**Keywords:** avian influenza, disease hotspot, mathematical epidemiology, SIR model

## Abstract

Influenza, categorized as one of the emergent infectious diseases, presents a substantial public health concern due to its capacity to trigger extensive epidemics and global pandemics. Every recent pandemic of human influenza has been attributed to avian influenza viruses (AIVs), underscoring their pandemic potential and the associated public health risks. Interestingly, there remains a significant knowledge gap concerning the mechanisms that sustain the survival and proliferation of these viruses within their natural avian reservoirs. Migratory waterfowl, in particular mallard (*Anas platyrhynchos*), plays a crucial role as potential reservoirs, facilitating the spread of the virus through their migratory patterns. In order to better understand the factors that contribute to AIV spillover and hotspots, we built a mechanistic transmission model and investigated the likelihood of an AIV hotspot for the animal‐to‐human spillover of the virus in a region in Kansas. Our findings challenge the notion that the size of the overall mallard population is a reliable predictor of spillover hazard. To study this aspect, we systematically evaluated weekly trends in both the total population density and the total infected population density. In more than 70% of the locations studied, these two indicators showed periods with opposite trends. This conclusion stresses the importance of developing and calibrating compartmental models that can capture the diffusion of the virus within the reservoir and its spatiotemporal distribution due to animal movement.

## 1. Introduction

The occurrence of newly emerging and re‐emerging diseases has been demonstrated to have increased over the past years and the complex structure of propagation of these diseases is attributed to constantly changing environmental and behavioral factors, such as climate change, urbanization, and globalization [1, 2]. Avian influenza, one of the emerging infectious diseases, poses a significant public threat due to its ability to cause widespread epidemics and pandemics around the world, along with devastating health consequences among human and animal populations [3–5]. For example, the highly pathogenic subtype of the avian influenza virus H5N1 (HPAI H5N1) has been documented to circulate worldwide, with reported mortality rates of up to 100% in poultry flocks [6].

The substantial mortality rates and frequent disease outbreaks present significant challenges to the sustainability of poultry as primary sources of food and economic stability in both industrialized and less‐industrialized communities [7–10]. The consequences can be particularly severe for households in terms of both nutrition and economic impact, especially in rural areas where households frequently largely depend on their backyard poultry [11–13]. During the period 2004–2006, poultry losses in H5N1‐affected countries amounted to nearly 1% of GDP. Its impact reached 62 countries by June 2007, resulting in the death or slaughter of over 250 million birds and an estimated economic impact exceeding $12 billion, mostly due to disruptions in global food prices and poultry product supply [14–17]. More recently, between October 2021 and November 2022, over 5.5 million birds were put to death as a result of the continuing outbreak of bird flu in the United Kingdom. About 40 million animals have died as a result of the 2022 avian influenza virus (AIV) outbreak in the United States, and the financial implications have ranged from $2.5 to $3 billion [18]. In addition to causing enormous losses to the poultry industry, AIVs have also caused mass deaths among wild birds, which has raised serious concerns about the preservation of wildlife and represents an emerging disease [19].

In North America, the ongoing H5N1 outbreak that began in 2022 has demonstrated an unusual trajectory: it has infected domestic poultry, wild birds, marine mammals, and even dairy cattle—something not seen before 2024 [20]. As of mid‐2025, nearly 1000 U.S. dairy herds across 17 states have confirmed infections [21], and over 160 million domestic birds have been affected. Although the United States has recorded only three confirmed human cases in 2025 (it was 67 in 2024), the virus continues to circulate across species boundaries, underscoring its zoonotic potential [22]. While human‐to‐human transmission has not been observed and the risk to the general public is still considered low, these developments clearly show the urgency of integrated, cross‐species surveillance systems and mechanistic modeling tools capable of anticipating spillover risks during migratory bird returns.

Although incidents of human‐to‐human infection with AIV have been reported, the majority of human cases of AIVs are caused by direct virus transmission from infected birds to humans [23, 24]. AIV H5N1 was first discovered in humans in 1997 in Hong Kong and after the first‐ever case, it has killed nearly 60% of those infected by that outbreak [25, 26]. The situation did not improve over time, and more than 800 individuals contracted H5N1 between 2003 and 2016, and the death rate was higher than 50% [27]. In the past few years, there have been several documented sporadic outbreaks; most of these have been located in the United States, Mainland China, East Asia, and a few smaller outbreaks in Europe [28]. Since 2020, there have been 67 documented cases among humans of the highly pathogenic avian influenza (HPAI) variant, resulting in 22 fatalities, along with 48 cases of the low‐pathogenicity avian influenza (LPAI) variant, with one reported fatality [29]. Due to ongoing outbreaks, recorded cases, and the zoonotic potential of certain dangerous variants, AIV is associated with a serious pandemic risk and is of great concern for global public health [28].

Given the substantial consequences for both humans and animals, numerous works have been conducted on the transmission dynamics of AIV [30–35]. In light of the complexity, mathematical models, in particular mechanistic models, provide useful tools for understanding the dynamics of epidemic diseases and for synthesizing data to comprehend underlying mechanisms that lead to propagation of diseases [36, 37]. To this end, researchers have aimed to quantify transmission rate values primarily within flocks. This helps to determine the time of virus introduction into a flock, estimate the proportion of animals necessitating vaccination, and assess the effectiveness of intervention strategies [38–41]. While considerable attention has been dedicated to researching AIV transmission among poultry animals, the disease spread among wild birds remains relatively understudied. AIVs easily spread across regions through migratory wild birds and international trade in live poultry, facilitating rapid disease transmission [23]. In fact, migratory waterfowls act as reservoirs, facilitate the spread via migration, and it is documented that more than 100 bird species are hosts for AIV [42–44]. The challenging task of estimating the capacity for infected hosts to carry the virus, especially over long‐distance movements, complicates our understanding of how pathogens can rapidly spread [45, 46]. In addition, researchers predominantly focus on HPAI over LPAI considering the severity of the illness, despite the virus’s ability to mutate and potentially cause significant morbidity [47]. Constructing a realistic model is highly complex, as it would need to encompass transmission within and among multiple potential species of wild hosts, including migratory species, that inhabit seasonally forced environments [34, 48]. Seasonal fluctuations, spatial interconnections, and host population migratory patterns affect the dynamics of AIV transmission [49]. These elements are frequently oversimplified in traditional disease models, which results in a limited ability to forecast AIV dynamics and potential spillover and outbreaks. Therefore, recent studies point out the importance of addressing these limitations through more advanced formulations. Spatiotemporal reaction–diffusion models have demonstrated how spatial heterogeneity alters epidemic thresholds and propagation dynamics [50]. Multidelay avian influenza models have revealed how Hopf bifurcations give rise to oscillatory disease dynamics and how optimal control interventions can stabilize outbreaks [51]. Fractional‐order SIR frameworks with varying population sizes have shown how memory effects and nonclassical temporal responses influence transmission [52]. Alongside past research on multispecies models [53], optimal control strategies [54], and density‐dependent diffusion [55, 56], these studies collectively demonstrate the variety of mathematical tools currently being used to study avian influenza and stress the significance of taking spatiotemporal and parametric complexity into account when designing models.

For a better representation of the disease propagation, considering spatial dynamics is also necessary. This level of detail has the potential to improve the understanding of the spread of the influenza virus between different locations, identify the development of local hotspots, and assess the possibility of cross‐species transmission, particularly considering the risk of wild birds distributing viruses between countries or even continents [44, 57]. The literature discussed so far mostly assumes that the spatial diffusion rates of the bird population are either constant or depend exclusively on the location (i.e., not on time) [55, 58–62]. A notable exception is the work by Liu et al. who used a diffusion rate represented as a continuous, positive, and periodically varying function [55]. However, this oversimplifying assumption does not accurately reflect real‐world conditions, particularly when dealing with infectious diseases [56]. Hence, one main goal of this paper is to introduce nonlinear density‐dependent diffusion terms influenced by seasonal variations and specific locations, with the aim of improving the accuracy of animal movement estimation. We seek to address the research gap in models of the various details of influenza dynamics in wild bird populations, particularly mallards, by developing an improved temporal–spatial compartmental framework with time‐dependent diffusion. The goal of this research is to contribute to a deeper understanding of the dynamics of infectious diseases between wildlife and humans, support public health efforts, and provide practical insights into preventive measures.

## 2. Methods

### 2.1. Study Sites and Data

**Figure 1:**
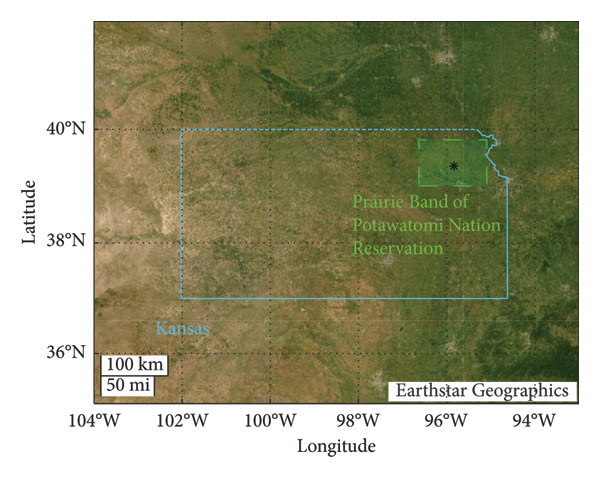
The map depicts the geographical boundaries of the state of Kansas, which are represented by the solid lines. The dashed lines on the map show the study region (Region 1), specifically encompassing the Prairie Band Potawatomi Nation Reservation.

We focus this study on data located near the territory of the Prairie Band Potawatomi Nation (39.3611°N, 95.8354°W), home to an indigenous community located in the U.S. state of Kansas (Figure 1). The selection of this particular region for our study is due to the fact that such diseases tend to exhibit a higher incidence among under‐represented and underserved communities, imposing a disproportionate burden on minority communities [63, 64].

In addition to this primary study region, our analysis includes eight additional sites across North America. These locations were deliberately selected to represent a broad range of climatic conditions, urbanization levels, animal population densities, and bird biodiversity (see Section 2.3). This allowed us to capture a diverse range of conditions known to influence AIV transmission and host population dynamics. Moreover, this broader analysis scope enables us to evaluate the generalizability of our findings and test the robustness of the proposed model across varied real‐world conditions.

Across all study regions, mallards *Anas platyrhynchos* represent the predominant species among waterfowls, and this species is also recognized as the major primary reservoir for AIVs [65, 66]. Due to their vulnerability, their migratory patterns, and their frequent encounters with different bird populations, mallards are regarded as having a crucial role in the maintenance and transmission of AIVs and are the focus of this study [67].

For each site, to estimate the total mallard population, the weekly number of birds present in each of 441 grid cells, measuring 2.96 × 2.96 km^2^, was collected from the eBird database, which provides comprehensive bird species abundance data worldwide for the year 2020 [68]. This is the richest available time series of bird species abundance over North America, where “abundance” is used as a technical term to indicate the number of animals in each grid cell [69]. For some cells, no observations were available in the database. Even though the absence of observations does not necessarily imply the absence of birds in those locations because data has to be manually submitted by an eBird user for it to appear on the database, places with no observation were assigned 0 counts for our purposes. To ensure meaningful comparisons across regions, we filtered for sites with sufficient temporal resolution (i.e., at least 30% nonzero weekly values per grid cell across the year). This filtering process yielded the nine regions used in our study. While Region 1 was selected for its relevance to our motivating application, the remaining eight were chosen based on coverage completeness in the eBird dataset, along with their diversity in climate, urbanization, animal density, and bird biodiversity (see Section 2.3).

Although the eBird database provides data only for year 2020, we extended it to encompass the subsequent year (2021) under the assumption that observed trends would remain consistent, thereby simulating a 2‐year time frame for all nine locations using the same modeling framework.

### 2.2. The Model

Our proposed compartmental model assumes mallards exist in three potential disease states: (S) susceptible, (I) infected, or (R) removed/recovered. The SIR model consists of the following system of partial differential equations:

(1) ∂S∂t=bN−μS−βSI+εR+dSΔS,

(2) ∂I∂t=−μI+βSI−γI+dIΔI,

(3) ∂R∂t=−μR+γI−εR+dRΔR,

(4) Nt=St+It+Rt,

where *b* is the birth rate, *μ* is the death rate, *β* is the transmission rate, *γ* is the recovery rate, *ϵ* is the loss of immunity rate, *d*_*S*_, *d*_*I*_, and *d*_*R*_ are the diffusion rates for each disease state, and *N* (*t*) is the total number of mallards at time *t*, as provided by the eBird data. It is assumed that the diffusion constants are equal across all compartments (*d*_*S*_ = *d*_*I*_ = *d*_*R*_ = *d*). We assume that these rates are equal because there exist no data to suggest otherwise. The parameters *β*, *γ*, *b*, *μ*, and *ϵ* are constant across all 441 grid cells and over time (i.e. they are not time‐dependent). Instead, the diffusion coefficient (*d*) is a function of time and space.

Several assumptions have been made for our model. First, it is assumed that AIV transmission occurs in a density‐dependent manner, reflecting the notion that transmission is influenced by population density, rather than frequency‐dependent transmission, because it was already determined that this modeling approach is more appropriate for mallards [70]. Second, we did not include the possible interactions with other host species. In reality, other bird species interact with mallards in their shared environment, and this can affect how diseases spread. Despite the fact that other species are outnumbered by mallards numerically, their influence on the dynamics of AIV transmission should not be neglected [71].Third, considered to be born disease‐free, making them initially susceptible to the AIV. Moreover, the boundary conditions represent the population dynamics at the edges of the study area as provided by eBird data.

For the first week, at each location, the initial condition *N*_0_(1) is set equal to the number of birds *N*_*e*Bird_(1) reading from the eBird database for week 1 of year 2020. For the other compartments, we set *S*_0_(1) = 0.3*N*_0_(1),  *I*_0_(1) = 0.4*N*_0_(1), and *R*_0_(1) = 0.3*N*_0_(1) as initial conditions. We tested also four other sets of initial condition distributions, and we confirmed that this choice does not affect the results after a very brief initial transient, which is irrelevant because all conclusions are drawn based on the results obtained for the second year of the simulation. From these initial conditions, the proportion of birds in *S*, *I*, and *R* is propagated according to the dynamics in (1)–(4) to compute their values at the end of the first week: *S*(1), *I*(1), and *R*(1). Then, these values are rescaled to match the total number of birds observed at the beginning of the following week in eBird, *N*_*e*Bird_(2). In other words, the initial conditions for a generic week are set as

(5) N0t+1=NeBirdt+1,

(6) S0t+1=St·NeBirdt+1Nt,

(7) I0t+1=It·NeBirdt+1Nt,

(8) R0t+1=Rt·NeBirdt+1Nt.

These steps are iterated to cover two years, until week 52 of year 2021.

**Figure 2:**
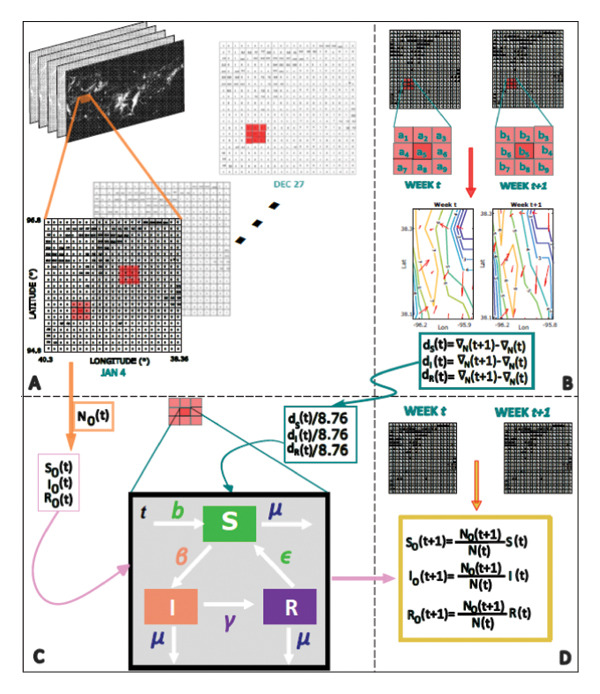
Schematic representation of the SIR model of AIV in a mallard population. Panel A: The abundance data for mallards is obtained from the eBird database for the year 2020. The data undergoes cleaning to convert all NA values to 0. Panel B: The gradient is computed using the central difference method for each location and time. Then, the difference between consecutive gradients divided by the grid’s area provides the spatial diffusion rate for each compartment. Panel C: The temporal dynamics of each cell, where mallards are assumed to be born susceptible (*S*), get infected (*I*), and can recover from infection (*R*). Panel D: Population of each compartment for the next time step is rescaled by multiplying the results of the simulation by the ratio of population at each consecutive time instant.

A comprehensive overview of the fundamental components that constitute this model is provided in Figure 2. The diffusion parameters, which measure the speed at which the mallard population spreads through space, indicate that this dispersion rate is not uniform. We used central differences to compute gradients ∇_*N*_(*x*,  *y*,  *t*) for all points along latitude and longitude within the region. The difference between each consecutive gradient in time determines the diffusion rate. A scale factor is used to standardize the unit of measurement by the surface area of each cell. This scale factor was set to 8.76 km^2^ (2.96^2^ km^2^), for each cell which is the resolution of the data extracted from eBird. This approach helps us understand how these variables change both spatially and temporally.

### 2.3. Numerical Simulations Parameters

The initial conditions have already been discussed in the previous section. The other model parameters, as specified in (1)–(3), are detailed in Table 1. Transmission rates of AIV in wildlife populations remain largely uncertain. Thus, we have selected a value for *β*, ensuring that the basic reproduction number of the virus (*R*_0_) spans from 0.8 to 8.0 [33, 72, 73]. Specifically, the transmission rate is selected such that *R*_0_ equals 2, which is determined by the equation = *R*_0_·*γ*/*N*, where *γ* is the recovery rate and N is the average number of mallards in a given region.

**Table 1:** Standard parameter values for mallards.

Symbol	Definition	Ave. value	Max. value	Min. value	Units	Source
*b*	Birth rate	0.0385	0.0481	0.0289	weeks^−1^	[74]
*μ*	Death rate	0.0096	0.0072	0.012	weeks^−1^	[74]
*β*	Transmission rate	1.5649	1.9561	1.1737	birds^−1^ weeks^−1^	[75]
*γ*	Recovery rate	0.875	1.094	0.656	weeks^−1^	[45]
*ϵ*	Loss of immunity rate	0.0385	0.0481	0.0289	weeks^−1^	[71]

### 2.4. Metrics to Evaluate Association Between Total and Infected Populations

To quantitatively measure the alignment or divergence between *N* (*t*) and *I*(*t*), four metrics are defined in (9)–(12). These metrics are the ratio of analysis time steps in which the total population exhibits a rising trend while the infected bird density shows a declining trend (*A*_1_); the ratio of analysis time steps in which the infected bird density exhibits a rising trend while the total population shows a declining trend (*A*_2_), the ratio of analysis time steps in which one set exhibits a rising trend while the other shows a declining trend (*A*_3_ = *A*_1_ + *A*_2_), and the proportion of locations with at least one pair of gradients having opposite signs during the second year of simulations (*A*_4_). These metrics were introduced to provide a structured and quantitative assessment of the relationship between total mallard density and the dynamics of infected bird populations.

(9) A1=∑i=1K∑j=1L1Nij is increasing and PIij is decreasingK·L,

(10) A2=∑i=1K∑j=1L1Nij is decreasing and PIij is increasingK·L,

(11) A3=∑i=1K∑j=1L1Nij and PIij have opposite signsK·L,

(12) A4=∑i=1L1at least one pair of Ni and PIi have opposite signsL,

where *K* is the total number of analysis time steps for the entire region, *N*_*i* *j*_ is the change in total population of mallards in time *i*, and at location *j*, ${\left(P_{I}\right)}_{ij}$ is the change in proportion of infected mallards at time *i* and location *j*, *L* is the total number of locations that have at least one divergent entry across the studied period, and *condition* is equal to 1 if *condition* is true and equal to 0 otherwise. When defining these metrics, we specifically omitted locations that lack any records of mallard observations throughout the entire time history.

### 2.5. Versatility of the Framework Across Various Contexts

We carried out analysis in various locations with varying climatic conditions, levels of urbanization, animal populations, and biodiversity of bird species in order to evaluate the ability to extrapolate our findings to various geographic, environmental, and ecological contexts. These regions were carefully chosen to span a broad range of important features that can potentially produce different results. For the sake of simplicity and relevance to our project, this additional analysis focused on North America. The average values of parameters listed in Table 1 were used, and we compared the metrics listed in (9)–(12).

**Figure 3:**
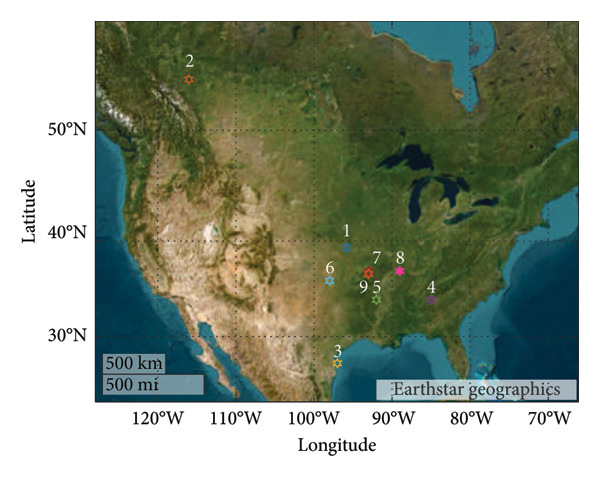
The locations of nine studied regions are displayed together with their corresponding identifiers. Note that region #1 corresponds to the study discussed in the previous subsections.

It should be noted that the primary area of interest in this research (Region 1) falls within the mixed‐humid climate category, has a small metropolitan level of urbanization, a medium‐sized animal population, and medium biodiversity among birds. Based on these specific characteristics, we have selected 8 additional locations, which are visualized in Figure 3.

For climate conditions, three categories were considered: very cold, mixed‐humid, and hot‐humid [76]. Three categories were considered for the level of urbanization: big metropolitan areas, minor metropolitan areas, and micropolitan regions [77, 78]. The density of animals was also taken into account, and it was divided into three categories: low (0–1), medium (1–50), and large (50 and above). Finally, the biodiversity of birds was evaluated and divided into three levels: low, medium, and high [79]. These classifications were made to take into consideration regional differences in the variety of bird species.

### 2.6. Sensitivity Analysis

#### 2.6.1. Parameter Ranges and Sampling Strategy

In this study, we conducted a sensitivity analysis on all the fixed model parameters described in Table 1 to evaluate model robustness. Since some parameters used in this study (e.g., infection rate, *β*) are deterministic and do not have any assigned specific probability distributions, we introduced variability by assigning uniform distributions that allow for a ±25% variation around their average values given in Table 1. Thus, the minimum value for each parameter is set at 75% of its mean value, and the maximum value of each parameter becomes 125% of its assigned value.

We first carried out parameter‐specific tests to see the impact of each parameter individually. To do so, we varied the value of each parameter (*b*, *μ*, *β*, *γ*, *ϵ*) in 1000 regularly spaced increments between its maximum and minimum values. This procedure allowed us to isolate the effect of individual parameters on both the magnitude and trajectory of infection dynamics.

Second, we examined interactions between parameters by varying them simultaneously. To do this, we generated 1000 parameter sets covering the joint uncertainty space of (*b, μ, β, γ, ϵ*). Our procedure was designed such that the relative position of each parameter within its range was aligned across all parameters. In other words, for each set the same scaling factor in the range (0.75, 1.25) was applied to the mean value of all five parameters. This aligned sampling strategy effectively generates parameter vectors along the “diagonal” of the multidimensional uncertainty space, capturing scenarios in which all biological processes are simultaneously slower (e.g., lower infection, recovery, and birth rates) or faster (e.g., higher infection, recovery, and birth rates). Such correlated shifts are more biologically meaningful than fully independent sampling, since natural conditions (e.g., seasonality, habitat quality, or climate stressors) drive multiple demographic and epidemiological processes in the same direction at once.

#### 2.6.2. Temporal Resolution Tests

To test the robustness of our findings against temporal resolution, we performed downsampling using eBird data. We considered three cases: observations recovered every week (reference), an alternate scenario with data observed every other week, and a more spaced‐out scenario with data observed every 4 weeks. This test probed the sensitivity of the model to data sparsity and examined whether the observed discrepancy in trends of between total and infected populations persists when fewer observations are available.

## 3. Results

The key results from our analyses show that (1) relying only on the total mallard density as a proxy for assessing the spillover hazard is inadequate; (2) a thorough calibration of some of the model parameters is crucial to have accurate quantitative assessments and regular data collection for mallards is necessary for higher temporal resolution, but the qualitative conclusion mentioned in the previous point remains valid for all realistic parameter sets; (3) the introduced framework can be expanded to other geographic locations to have specific results, but the presented findings appear to be general.

### 3.1. Relationship Between Total and Infected Population Sizes

**Figure 4:**
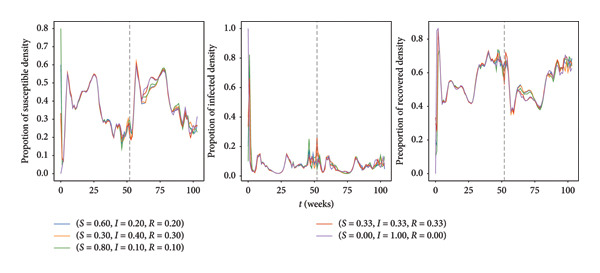
The choice of different initial distributions for the three compartments has relatively minor effects. Specifically, the behavior of infected bird density exhibits similar patterns for the various initial distribution scenarios after the initial 52‐week period. This observation indicates the robustness and stability of the study’s outcomes over an extended time frame, regardless of the specific initial conditions.

We performed a small sensitivity analysis to explore how the initial distribution of mallards across different compartments impacts the overall outcomes of our study. Due to the inherent uncertainty regarding the initial distribution, several options were investigated to see the magnitude of their effects. While there exist infinitely many possibilities for the choice of initial distributions, the selected ones are determined by common practices found in the current literature [80, 81]. Figure 4 reveals that the choice of initial distribution leads to a minor influence during the initial phase. It is important to note that the (0.0, 1.0, 0.0) setup reflects the situation where all individuals are infected at the beginning of the analysis. This particular configuration was intentionally included in the analysis, despite the fact that it is highly unrealistic, to explore a bound. Even in this unusual setup, the effects of the initial distribution diminish quickly. The overall trend in the long run shows that these different initial conditions tend to converge and align, demonstrating a good level of agreement. Yet, a metric cut‐off was established at 52 weeks, designating the initial year as a transitional period. Our focus is on analyzing the trends and results during the subsequent (i.e., second) year.

A study of the variation over time in the density of infected mallards (which actually constitute spillover hazard) and the total density of mallards at each location was conducted. The time histories computed at a representative location in Region 1 are presented in the top panel of Figure 5 and serve as an illustrative example of the alignment or contrast between the two trends under examination. The blue‐ and red‐shaded regions in this panel, shaded areas in Figure 5, highlight weeks with an opposite trend between the total mallard population and density of infected mallards at the considered location. The dynamics of infected bird populations and the total mallard density are not consistently moving in the same direction. In fact, the trends here diverge in 17 instances over the course of a year (52 weeks), accounting for approximately 32.7% of the total year duration. Moreover, the top panel shows that the fluctuations are not diverging just because of some temporal lag; they have very different trends. The bottom‐left panel of Figure 5 displays the temporal evolution of the susceptible, infected, and recovered compartments as proportions of the total population. Meanwhile, the bottom‐right panel shows the raw mallard abundance values from the eBird database for the same location, capturing the seasonal trends in *N*(*t*) used to scale the compartmental outputs.

Figure 5(a) Density of infected birds, denoted as *I*(*t*) (orange), and total abundance of mallards, *N*(*t*) (gray), at a selected location in Region 1. Blue‐shaded intervals (*A*_1_) indicate periods where *N*(*t*) increases while *I*(*t*) decreases; red‐shaded intervals (*A*_2_) indicate the opposite. These intervals are used to compute metrics *A*_1_ and *A*_2_ as defined in Section 2.4. (b) Time evolution of the SIR compartments: susceptible (*S*, blue), infected (*I*, orange), and recovered (*R*, green), normalized by total population. (c) Raw mallard abundance data from eBird (*N*(*t*), gray) at the same location, plotted independently to visualize seasonal population trends.

**Figure:**
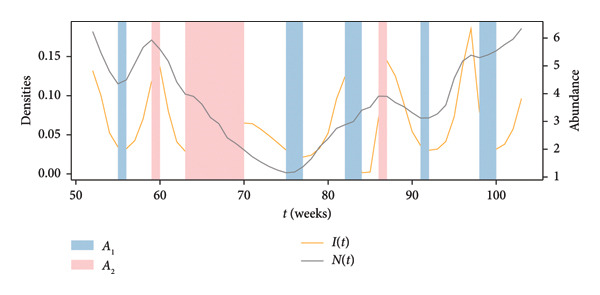
(a)

**Figure:**
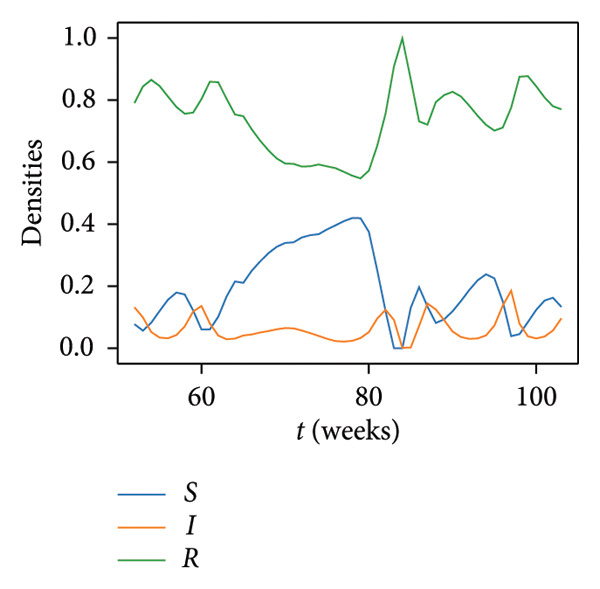
(b)

**Figure:**
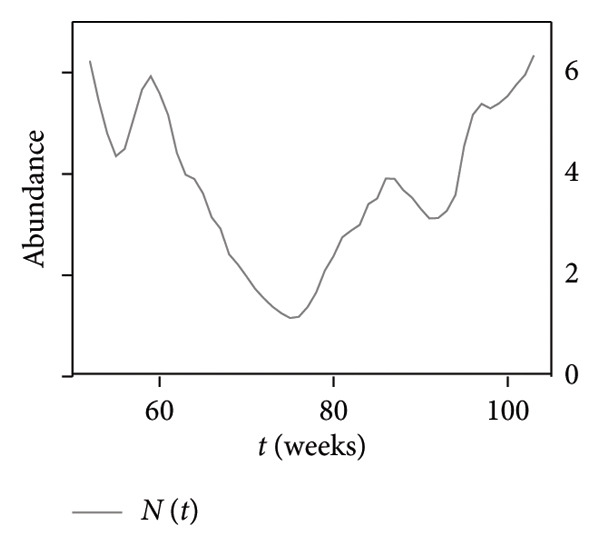
(c)

The metrics defined in Section 2.4 were also assessed for each location in the studied region, first for the entire time period and then just focusing on weeks 53–104, after the transient period is complete. The results for Region 1 are provided in Table 2. For the complete time history, we observe that approximately 8.0% (*A*_1_) of time steps show increasing total abundance and decreasing infection, while 11.9% (*A*_2_) of time steps show the opposite pattern. The combined measure of divergence *A*_3_ = *A*_1_ + *A*_2_ = 19.9%, indicating that nearly one‐fifth of the time steps reflect misaligned trends between total population and infection. Moreover, 81.9% (*A*_4_) of locations exhibit at least one instance of divergence, demonstrating the spatial prevalence of these opposite dynamics. Focusing on the post‐transient period (weeks 53–104), the values shift slightly: *A*_1_ increases to 8.5%, *A*_2_ drops to 10.3%, and A_3_ remains similar at 18.8%. Notably, *A*_4_ decreases to 71.6%, suggesting that while the phenomenon of opposing trends remains widespread, it is slightly less pervasive once the system stabilizes after the initial transient. These results confirm that the divergence observed at the representative location in Figure 5 is not an isolated occurrence but a persistent and widespread feature across the region, even after transient dynamics have settled.

**Table 2:** Metrics of the discrepancy between total and infected population for Region 1.

Metrics	*A*_1_	*A*_2_	*A*_3_	*A*_4_
Entire time history (weeks 1–104)	8.0%	11.9%	19.9%	81.9%
After transient (weeks 53–104)	8.5%	10.3%	18.8%	71.6%

At the representative location shown in Figure 5, we observe opposing trends between the total and infectious populations. Since *β*, *γ*, and *R*_0_ are constants, this phenomenon is driven by the diffusion terms. Notably, during weeks 65–70, the diffusion terms are positive, which directly contributes to the increase in the infected population by redistributing individuals across spatial areas. The variations in diffusion directly affect the local densities, leading to the observed behavior despite constant epidemiological parameters.

### 3.2. Sensitivity Analysis

**Figure 6:**
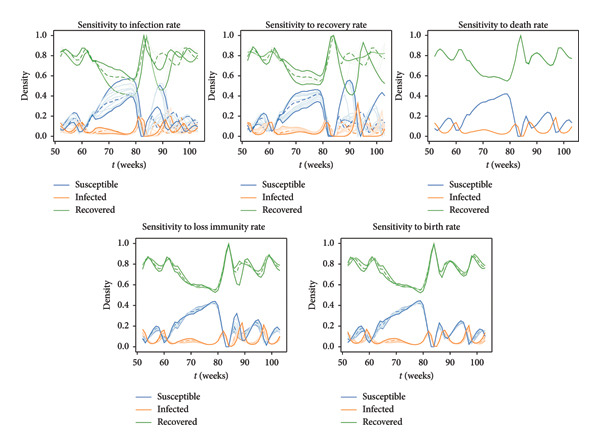
Effects of each parameter varied individually. All results are for a given location. The solid dark lines depict the results obtained by setting each parameter to its lower and upper bounds. The lightly shaded lines represent the results obtained by setting each investigated parameter to some intermediate values. Notably, in some cases, the results obtained with intermediate values of the parameters fall outside of the range determined by the results obtained with the minimum and maximum values of the parameters. The dashed lines correspond to the results obtained when the parameters take the average values specified in Table 1. The figure reveals that variations in birth rate, death rate, and loss of immunity rate lead to relatively minor effects compared to the other factors. In contrast, the ranges are much more expansive for the infection and recovery rates, signifying their greater influence on the system dynamics.

We conducted a sensitivity analysis to see the individual and compound effects of each parameter on the outputs of interest. Figure 6 visually displays the whole test findings for each specific parameter, giving a summary of how sensitive each output is to changes in specific parameters. The first observation is that the trend remains largely consistent in almost all cases. Figure 6 shows that the variations in birth rate and death rate have only minor effects and the trends are stable. However, the densities are highly affected by the recovery rate and, more importantly, infection rate.

**Figure 7:**
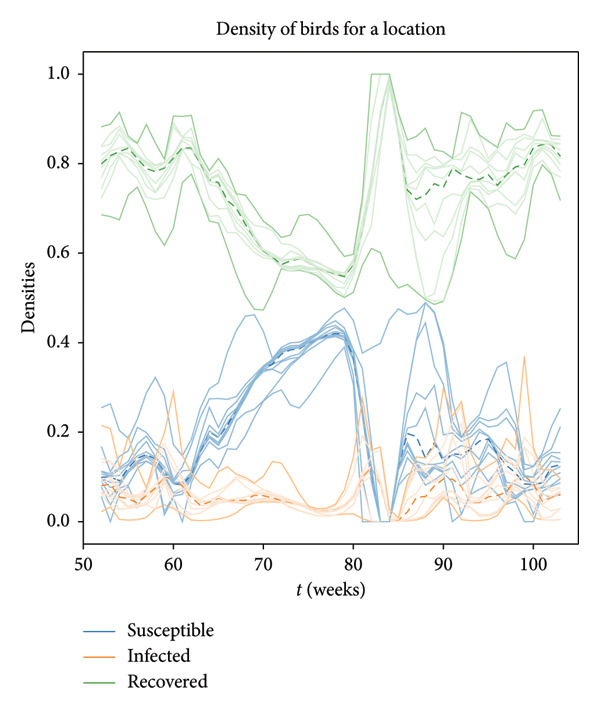
Proportion of susceptible, infected, and recovered birds from weeks 53–104 for the 1000 selected parameter sets. The dashed lines are those proportions that correspond to average values in Table 1. The opaque lines correspond to those proportions obtained when we select the minimum possible parameter values and the maximum possible values. The lightly shaded lines correspond to the other 997 combinations of parameter values. Each trajectory’s trend emphasizes the overall robustness of the approach.

Our study also includes an analysis of the compound effects resulting from the simultaneous change of all parameters in addition to the individual parameter assessments (Figure 7). We observed a good level of consistency in the general results previously identified in the individual effects analysis in Figure 6. The trends over time remain quite similar for all considered parameter sets.

**Figure 8:**
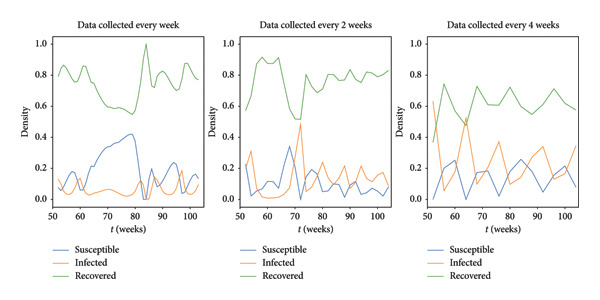
Densities under different temporal resolutions for a location.

We performed a similar analysis with various temporal windows, recognizing that the highest resolution may not always be achievable. The findings in Figure 8 indicate substantial differences, even when dealing with biweekly observations. The model becomes unstable and cannot capture the general trend when total population data is gathered only once every 4 weeks.

### 3.3. Ability to Extend the Results to Other Situations or Contexts

Table 3 collects the values of the discrepancy metrics in (9)–(12), focusing only on the results after week 52 to eliminate the transition period. While the metrics’ values may vary, the opposite trend between *N* (*t*) and *I*(*t*) is observed for each geographic region considered. It is clear that contrasting trends occur in each study region in at least 10% of the covered locations. This percentage increases to as much as 72% for Region 1, which is the focus of our study. The broad range of these values shows the substantial impact of the selected factors in our analysis.

**Table 3:** The values of metrics for different classes.

Region	Climate	Level of urbanization	Animal density	Biodiversity	*A*_1_	*A*_2_	*A*_3_	*A*_4_
1	Mixed‐humid				**8.5%**	**10.3%**	**18.8%**	**71.6%**
2	Very cold	Micropolitan	Medium	Medium	1.0%	0.6%	1.6%	10.2%
3	Hot‐humid				6.3%	5.6%	11.9%	49.3%
4		Large metropolitan			3.8%	2.4%	6.2%	29.0%
1	Mixed‐humid	Small metropolitan	Medium	Medium	**8.5%**	**10.3%**	**18.8%**	**71.6%**
5		Micropolitan			0.9%	1.9%	2.8%	22.5%
6			Low		7.6%	6.7%	14.3%	63.9%
1	Mixed‐humid	Micropolitan	Medium	Medium	**8.5%**	**10.3%**	**18.8%**	**71.6%**
7			High		1.8%	1.0%	2.8%	14.3%
8				Low	6.5%	5.0%	11.5%	52.3%
1	Mixed‐humid	Micropolitan	Medium	Medium	**8.5%**	**10.3%**	**18.8%**	**71.6%**
9				High	4.9%	3.0%	7.9%	41.3%

*Note:* Bold rows correspond to the primary focus of the study of the region.

## 4. Discussion

The primary finding of this research challenges the notion that the total population density of mallards alone is a sufficient indicator of the total population of infected mallards. In other words, we confuted the hypothesis that the percentage of infected individuals remains relatively constant over time and space, and therefore the total number of individuals can be used as a proxy to determine areas and weeks of the year at highest risk. Not only the percentage of infected mallards is not constant, but even the trends of population size and infected individuals are different. By examining these trends at a weekly resolution, the study identifies an interesting pattern: at approximately 70% of the locations in the study region, the trends in total population density and total infected population density are opposite to each other. This discrepancy implies that while the total population density is decreasing, the density of infected individuals may be increasing. Notably, in regions where spatial diffusion is more pronounced, this contrasting trend becomes even more apparent. If the hypothesis were correct, then it would have been possible to avoid complex compartmental models to make decisions on policies, models of the general bird population would be sufficient. But the results presented in this paper emphasize that this is not possible, and a compartmental model with spatial diffusion terms is necessary to have reliable snapshots and forecasts of the density of infected animals, to drive effective policies. While the analyses presented in this manuscript provide useful evidence, the conclusions are not entirely surprising, because the model of transmission in the zoonotic niche is fundamentally complex, it uses nonlinear terms to account for the infection dynamics and the spatial effects.

These observed patterns and their discussion are not exclusive to the specific study location. To confirm this point, we conducted similar analyses in various alternative regions (Figure 3). Table 3 reveals that despite differences in location characteristics such as climate, urbanization level, bird density, and biodiversity, the metrics that capture discrepancy between total population trends and infected individuals trends remain relatively consistent. Consequently, it is not advisable in general to directly extrapolate the total population density of birds to estimate the total density of infected birds. Future research should aim to acquire more data on infected birds through serological studies to further validate the findings presented in this study.

Another observation is the presence of multiple local maxima in infected mallards’ density, seen in Figure 5. This pattern is prevalent across a majority of locations in the study region. For comparison, seasonal human influenza in temperate regions typically shows a single winter peak, and bimodal (semiannual) patterns with two peaks per year have been documented in some tropical and subtropical settings [82–84]. Moreover, during the 2009 influenza pandemic, several countries, including the United States, encountered two distinct waves of infections, possibly influenced by vaccination rates and school seasons [85, 86]. However, in our results, we observe more than two peaks, which shows the crucial significance of wildlife surveillance. Public health authorities need to remain vigilant and gather continuous observations on wildlife populations. Such efforts are essential for early detection as well as for implementing proactive measures to inhibit potential spillover events, even after the initial wave of infections.

Serological studies indicate that the prevalence of AIV infection and antibodies against AIV in wild waterfowl tends to be high during late summer, before fall migration, corresponding to elevated waterfowl densities and a higher proportion of juveniles within populations [87–91]. In parallel to this statement, our results demonstrate that during late summer (week 83), the vast majority of the mallard population consists of individuals who have been infected and recovered, thus possessing antibodies against AIV. This alignment serves as a form of validation for our results, providing a confirmation of our findings.

Moreover, the objective of our sensitivity analysis was to evaluate the effects of single and multiple parameter changes on outputs of interest, namely the proportions of susceptible, infected, and recovered individuals relative to the whole population. Within their designated ranges, the birth and death rates showed only modest effects (Figure 6). On the other hand, population densities were affected by the rates of infection and recovery in a more substantial way, demonstrating their rapid effect on disease dynamics (Figure 6). The birth rate and death rate capture slow processes in population dynamics, so they contribute to changes in the total population size over longer time scales. Their variations may therefore have less of an immediate effect on the dynamics of the spread of infectious diseases. Conversely, as infections and recovery occur more quickly, they have more rapid effects on the population.

Our sensitivity analysis suggests that future studies should collect data to improve estimating the rate of infection and recovery in wild bird populations. The broad range observed in our results is due to uncertainties in these rates, emphasizing the urgent need for more accurate estimations. Beyond individual evaluations, we also explored compound effects resulting from simultaneous parameter variations. When all parameters are changed at once, the influence the dynamics of the system becomes slightly more pronounced (Figure 7). This confirms that it is important to take compound effects into account in order to have a thorough understanding of system behavior. In addition, our study was expanded to include various temporal resolutions. The results show that accuracy is lost when data is only gathered once every 4 weeks. These findings highlight how crucial it is to use frequent data gathering and high temporal resolutions in order to provide precise and dependable insights into the dynamics of infectious diseases. While it is practically challenging to collect this data frequently, our results show the value that such data collection has.

We had to make several assumptions in order to build relatively basic models that would allow thorough testing of a set of proposed drivers for AIV infection dynamics. The challenges presented by the availability of specific data and the need to find a balance between model complexity and practicality led to a number of assumptions. For example, the absence of historical information on the mallard population density was overcome by assuming that the seasonal trends in the 12 months of observations repeat identically year after year, which in the context of nonstationary climate may not be true. Severe climate changes can have a big impact on animal behavior and population dynamics [92, 93]. Additionally, the time dependence of certain parameters, such as the birth rate, death rate, transmission rate (*β*), recovery rate (*γ*), and loss of immunity rate (*ε*), was not explicitly included in our model. Although these parameters likely vary over time due to seasonal and environmental factors, we used constant values throughout the simulation to maintain model tractability and due to the limited availability of time‐resolved data. However, some time‐dependent effects can be implicitly represented through the computation of time‐dependent spatial rates. We adopted the common practice of assuming similar diffusive constants for the three compartments [53, 54, 56]. While many models adhere to this simplification, it is important to note that using different diffusive constants for each compartment could produce more accurate and nuanced findings in the future. Lastly, although randomness is a fundamental component of the problem, we concentrated mostly on its deterministic features for our study. AIV transmission in wild bird populations may be better understood in the future by incorporating stochastic aspects to account for the inherent variability and unpredictability in real‐world dynamics.

Taken together, these findings demonstrate several practical implications for wildlife surveillance and public health policy. First, our results demonstrate that relying solely on total bird population data (e.g., from citizen‐science databases such as eBird) is insufficient to accurately identify periods or regions of increased spillover risk. Models that distinguish between susceptible, infected, and recovered populations are needed to make informed decisions. Second, the observed opposing trends between total and infected bird densities across diverse ecological regions suggest that this phenomenon is generalizable and not location‐specific. Surveillance systems should therefore prioritize infection‐specific metrics over total abundance proxies. Third, the sensitivity of disease dynamics to infection and recovery rates points to a need for improved field data collection targeting these parameters, especially in undersampled regions. Finally, given the detection of multiple infection peaks per year and the loss of accuracy at coarser temporal resolution, we emphasize the importance of high‐frequency, spatially distributed monitoring programs. These are essential for early detection and for implementing proactive measures to prevent spillover events.

## Disclosure

This work is part of the activities of the *Center for Catastrophe Modeling and Resilience* at Lehigh University (https://catmodeling.lehigh.edu). A set of preliminary results on this topic was presented at in a poster entitled “Spatio‐temporal dynamics of avian influenza: understanding avian influenza transmission via mallard migration data” for an internal symposium at Lehigh University [94].

## Conflicts of Interest

The authors declare no conflicts of interest.

## Funding

The financial support from the US National Science Foundation through award PIPP–2200066 “Dynamics of Pandemic Spread and Prevention in Indigenous Communities” is gratefully acknowledged. Partial support from Lehigh University through the “Research Futures: Major Program Development” and the “Research Futures: Special Seed Funding Opportunity” grants is gratefully acknowledged.

## Data Availability

All data used for this research have been obtained from public sources cited in the manuscript. All code and data used in the analyses are available in a Figshare repository https://figshare.com/s/517b982e3a2af4f07fed.
